# Patients’ and Doctors’ Perceptions of a Mobile Phone–Based Consultation Service for Maternal, Neonatal, and Infant Health Care in Bangladesh: A Mixed-Methods Study

**DOI:** 10.2196/11842

**Published:** 2019-04-22

**Authors:** Mafruha Alam, Cathy Banwell, Anna Olsen, Kamalini Lokuge

**Affiliations:** 1 National Centre for Epidemiology and Population Health Research School of Population Health The Australian National University Canberra Australia

**Keywords:** mobile-based consultation, mHealth, remote diagnosis, referral

## Abstract

**Background:**

A mobile-based consultation service, or telehealth, can be used for remote consultations with health care professionals for screening, self-care management, and referral. In rural Bangladesh, where there is high demand for scarce male and even scarcer female doctors, remote consultations may help women seeking maternal and child health care. Aponjon is a mHealth service in Bangladesh that provides weekly voice or text messages to pregnant women, new mothers, and family members on various aspects of maternal, neonatal, and infant health. Subscribers can also access a dedicated 24*7 call center to discuss maternal, neonatal, and infant health or emergencies with medically trained doctors. The service provides advice, primary diagnoses, prescriptions, and referrals to subscriber callers.

**Objective:**

We investigated the Aponjon service to understand access, acceptability, usability, benefits, and challenges of a mobile phone-based consultation service.

**Methods:**

We conducted call log data analysis for September to November 2015 to understand how many unique subscribers accessed the service, who accessed the service, the geographical distribution of callers, and the purpose of the calls. We also conducted a qualitative exploratory substudy of eight married women and eight married men who were subscribers to and accessed the service during this time to understand their experiences. We interviewed 11 doctors from the same service who provided phone consultations to subscribers.

**Results:**

Approximately 3894 unique subscribers accessed the service for single or multiple consultations during the study period; 68.36% (2662/3894) of subscribers were from rural households, and 53.00% (2064/3894) of calls were made by pregnant women or new mothers. Approximately 96.08% (5081/5288) calls were nonurgent, 2.69% (142/5288) semiurgent, and 1.23% (65/5288) urgent. Almost 64.7% (134/207) semiurgent or urgent calls came between 8 PM and 8 AM. Callers found the consultation service trustworthy, cost-effective, and convenient. The doctors dispelled misconceptions and promoted good health care practices, regular health check-ups, and responsible use of medicine. They helped families understand the severity of sicknesses and advised them to seek care at health facilities for semiurgent or urgent conditions. The service lacked a pro-poor policy to support talk times of subscribers from poor households and a proper referral system to help patients find the right care at the right facilities.

**Conclusions:**

Although a regular messaging service is constrained by a one-way communication system, this service using the same platform, gave subscribers access to an abbreviated “consultation” with medical doctors. The consultations provided subscribers with valued medical advice and support, although they were limited in their population reach and their integration into the wider medical system. Further research is required to understand the impact of advice and referral, cost-effectiveness, and willingness to pay for mHealth consultation services, but this research suggests that these services should be supported or even expanded.

## Introduction

Telehealth, or mobile-based (mHealth), remote consultation service has the potential to improve maternal and neonatal health outcomes in developing countries such as Bangladesh, where mobile phone subscription rates are very high [1] and there is disparity within the health system in the distribution of trained doctors and qualified medical professionals in urban and rural areas [2,3]. Although Bangladesh has made significant improvements in reducing the maternal mortality ratio by 40% between 2001 and 2010 to 194 per 100,000 live births [4], it is still higher than in other South Asian countries [5]. The neonatal mortality rate (28 per 1000 live births) is also alarmingly high and contributes to almost two-thirds of the total under-five child mortality rate in Bangladesh [6]. Delay in seeking care from a health facility or formal health care provider is a major contributor to maternal and neonatal fatalities [7,8]. Research suggests that delay in seeking care is related to cost, inability to understand the severity of symptoms, and use of informal health care providers [4,7,8]. Furthermore, it is considered culturally inappropriate for women to seek maternal health care from male providers [9], yet female doctors are scarce in remote settings [2,9]. With the potential to address many of the barriers to seeking timely health care, a mobile-based consultation service could be used as a health promotion tool in raising awareness among women and their families in remote settings [10].

Mobile phone consultations between pregnant women and nurses at obstetric call centers were found to be effective in providing triage for determining emergency cases, helping patients manage nonurgent symptoms, and increasing efficiency in emergency departments at local hospitals in selected rural states of North America [11]. Another study in Australia found telephone helplines have potential for improving mother’s confidence and sustaining breastfeeding [12]. Evidence of interventions involving direct communication between client and provider is scarce in developing countries and is mostly reported in pilot studies [13,14]. Text and sometimes voice messages are the preferred interventions, seeking moderate positive behavioral outcomes in these settings [15,16] due to cultural contexts and operational challenges [13,17-20]. A pilot study in Southern Malawi reported a decrease in hospital attendance for child-related illnesses among an intervention group who were entitled to text or voice messages and a toll-free hotline service for maternal, neonatal, and child health care (MNCH) [21]. Community-skilled birth attendants who accessed toll-free mobile phones to consult with remote physicians were found to have improved confidence in treating and referring patients and gaining the trust of patients in a clustered randomized controlled trial in a subdistrict of Bangladesh [14]. Pilot interventions involving toll-free communication also allowed pregnant women to initiate calls to their local midwives regarding danger signs of pregnancy and delivery in Zanzibar [22] and Bangladesh [14]. Qualitative evidence from pilot projects in India suggested that 24-hour obstetric mobile phone-based helplines could potentially mitigate delays associated with seeking care, reaching proper health facilities, and receiving treatment [13].

Approximately 95% of urban and 84% of rural households in Bangladesh have access to a single mobile phone [1]. A study in a rural subdistrict reported an increase in household mobile phone ownership from 2% in 2004 to 81% in 2012 [23] indicating a rapid uptake of mobile phone technologies in a decade across the country [1]. Competitive pricing among telecom operators significantly dropped service prices for mobile phone owners and closed the “digital gap” of mobile phone ownership between low and high socioeconomic status (SES) households in Bangladesh; low SES households who were once behind the race were increasingly entering the mobile phone consumer market [24]. Understanding the multifaceted benefits of mobile phones in health care, the Government of Bangladesh adopted mHealth in its national Health Information Strategy to increase the performance of local facilities and access to health by all citizens [25].

In 2007, a private mHealth initiative in Bangladesh led to the development of a 24-hour helpline service run by the largest mobile phone operator, Grameenphone, in collaboration with Telemedicine Reference Centre Limited [26,27]. It initially charged 15 Bangladeshi taka (BDT; equivalent to US $0.18) for the first three minutes and then 5 BDT (US $0.06) for every subsequent minute [27]. In 2016, the helpline was made free to Grameenphone subscribers with additional benefits to support hospitalization costs under the new service name Tonic [28]. Following in the footsteps of Grameenphone, other telecom operators have introduced 24-hour remote counseling services to their subscribers [29]. There are also emergency mobile phone numbers provided for the district (*zilla*) and subdistrict (*upazilla*) public hospitals to reach physicians remotely free of charge [25]. The Ministry of Health and Family Welfare initiated a helpline service, “Shasthya batayan,” operated by a private call center, which provided citizens access to a doctor’s consultation and ambulance services through their mobile phones; customers were typically charged the usual call rates per minute [30]. However, there is a need for more scientific literature that correlates the effect of these teleconsultation services on the health outcomes of callers.

In addition to teleconsultation services, other mHealth initiatives in Bangladesh have involved the use of mobile phones to educate consumers about preventive health care behaviors and services. One such service, Aponjon, provides information to pregnant women and new mothers regarding pregnancy, delivery, and neonatal and infant health care since 2012 [31,32]. Registered female subscribers are entitled to receive two voice or text messages a week and an optional single message for their family member [31,32]. Based on feedback from subscribers who reported the need to talk to a medically trained doctor over the phone [31], Aponjon expanded its messaging service in 2013 to include consultations between patients and doctors [33,34]. The hotline service, 24*7, is available to Aponjon subscribers and uses a pool of medically trained, mostly female doctors [33,34]. The hotline service was critically vetted by experts in the field to ensure adherence with the national drug regulatory policy and counseling guidelines before being rolled out [29]. Subscribers are typically charged 2.3 BDT (US $0.03) per minute for the consultation service [34].

Although the Aponjon messaging service has been evaluated [32], the effectiveness of the medical consultation service has not. The voice and text messages show no or minimum effect on delivery and neonatal care practices among subscribers [32], although a quasi-experimental evaluation study [35] suggested that prolonged exposure to messages (6 months) can bring about behavioral changes. Another pilot study tested the feasibility of the nutrition counseling service among pregnant women and new mothers along with a mobile cash transfer system; however, the efficacy of the intervention is yet to be determined [36]. Given that the mobile-based consultation service involves the consumer calling (as opposed to being called) for advice and interaction with a health care professional, the acceptability of the service and effect on health behaviors are likely to be different to that of one-way messaging services. There is a dearth of literature on the effectiveness of mHealth consultation services for maternal and newborn health in resource-limited settings including Bangladesh [16,37]. The objective of this study is to describe the call volumes, the purpose of the calls, the experiences of subscribers, and the perception of doctors who provided consultations through the Aponjon service with a focus on acceptability, usefulness, and health promotion in the setting of Bangladesh.

## Methods

### Setting

Bangladesh is a country situated in South Asia with a population of more than 150 million in a land size of 147,570 km^2^, surrounded by India in the north, west, and east; Myanmar in the southeast; and the Bay of Bengal in the south [38]. Approximately 90% of the population is Muslim, 9% Hindu, and the remaining 1% are other religions such as Buddhists and Christians [39,40]. The economy of Bangladesh is rapidly progressing; the gross domestic product growth has been significant at a rate of 6% for the last decade, which has led Bangladesh to achieve lower middle-income country status in 2015 [41]. Administratively, the country is divided into 8 divisions, which are further divided into 64 districts (*zillas*) and 545 subdistricts (*upazillas*) [42]. The subdistricts, or *upazillas,* are further divided into urban areas (consisting of wards; *mohollas* or cluster of households make up each ward) and rural areas (consisting of union *parishads*; *mouzas* or clusters of villages make up each union *parishad*) [38].

Approximately 75% of the population resides in rural areas, although there is a trend of rapid urbanization [38]. The Ministry of Health and Family Welfare has the key responsibility to provide universal health coverage to urban and rural populations and operates a dual system of general health and family planning services through two Directorates General of Health Services and Family Planning in district hospitals, *upazilla* health complexes at the subdistrict level, union health and family welfare centers at the union level, and community clinics at the ward level [3]. Apart from government initiatives, private sector (formal and informal) and nongovernment organizations (NGOs) have established a network of facilities to provide health and family planning services [3]. There is no structured referral system or national health insurance policy; health services are mostly financed by each households’ out-of-pocket payment system [3]. Treatment facilities and medicine at public health facilities are subsidized; however, the quality of service has largely been criticized for the shortage and inequitable distribution of skilled health professionals in rural areas, lack of proper equipment, staff absenteeism, and governance issues [2,3]. Private facilities that have major state-of-the-art treatment equipment are urban-centric, costly, and remain out of reach of poor people [2,3].

### Aponjon Service

The Aponjon (meaning “the close or dear one” in Bangla) service was rolled out phase by phase across Bangladesh after being initially launched in 2012 [31,32]. The service underwent a year-long pilot before launching. Aponjon collaborated with the Government of Bangladesh and local NGOs to scale the service and recruit subscribers across the country [33]. The service is available through all telecommunication operators in the country [32].

The core service includes a messaging service—both interactive voice responses (IVRs) and text messages [31,32]. Typically, IVR messages are 1 minute long; the contents are structured around a female “doctor” (called *daktar apa*), who advises pregnant women, new mothers, and their husbands or caregivers on various aspects of pregnancy, delivery, postpartum and newborn care, illness symptoms, and nutrition [31]. The text messages were transliterated as Unicode and were not supported by basic mobile phones, resulting in it being popular among subscribers with higher education [31]. However, IVRs also became very popular among rural subscribers; 99% of them were receiving IVRs [32]. By mid-2014 the service had one million registered subscribers. Any pregnant woman can register in the service from 6 weeks of pregnancy if they have access to a mobile phone [31]. A potential subscriber may enroll her own phone or a phone that she shares with her husband or family members [31]. IVR messages are scheduled to be sent to subscribers according to their preferred time and days of the week to decrease the chance of them being missed [31]. Missed IVR messages can be retrieved by calling the service [31]. By the end of pregnancy, pregnant women are able to update their service to that for a new mother and receive messages for baby and mother until the child turns 1 year of age [31]. A new mother who did not receive the service for pregnancy can enroll in the new mother’s service [31]. Prospective subscribers are recruited to the service in several ways. The most popular way is through the thousands of community health workers [31] working under the flagship of different NGOs in Bangladesh [43]; they register interested women in the service during their monthly door-to-door visits for antenatal and postpartum care. Pregnant women and new mothers can also enroll with the help of paramedics or doctors at health centers [31]. The other option is to directly call the service for enrollment; the short code of the service had been popularized through advertisements in television, billboards, leaflets, and newspapers [31,32].

Since mid-2013, Aponjon subscribers can additionally talk to a doctor by dialing the service short code [34]. A pool of medically trained doctors, located in a central call center of Aponjon service in Dhaka city, handle calls regarding medical queries from subscriber women from across Bangladesh [34]. The purpose of the consultation service is to support subscribers to understand the severity of symptoms, help in decision making during emergencies, and improve understanding of maternal, neonatal, and infant health care practices at home through one-to-one consultation.

The Aponjon service has a pro-poor policy [31,32]; the socioeconomic data gathered during enrollment are assessed to consider the eligibility of marginalized subscribers to receive the messages free of charge. The rest of the subscribers pay 2.3 BDT (US$ 0.03) per message [32]. Aponjon subscribers accessing the service under any telecom operator will have similar pricing experiences. The “free of charge” policy is not applicable when subscribers access the consultation service; they pay the usual call rate for outgoing calls.

### Study Design

We conducted a mixed methods study. In September 2015, the service had approximately 1,135,839 subscribers from across Bangladesh. Anyone registered in the service could access the 24*7 consultation service. We analyzed the call log data of the consultation service. We also conducted qualitative interviews with subscribers who contacted the consultation service and with Aponjon doctors who provided consultations.

All participants provided written and verbal consent for this study. The study received ethics approval from the Science and Medical Delegated Ethics Research Committee, the Australian National University, along with permission from Dnet, the implementing agency of Aponjon in Bangladesh.

### Quantitative Data Collection

Each day, the Aponjon consultation service received an average of 150 calls from existing subscriber women’s cell numbers. All calls were logged on the service “customer relationship management” (CRM) database including date, time, callers’ mobile phone numbers, location, call duration, type of caller (subscriber woman/husband/family member), severity of the condition for which advice was sought (general, semiurgent, and urgent), and type of advice provided by the doctors. For the quantitative study, we retrospectively accessed the CRM database and extracted data for the period of September 1 to November 30, 2015.

### Qualitative Data Collection

All interviews were conducted between December 2015 and January 2016. For this study, we accessed the CRM database for calls made in the 3 months prior to data collection to reduce users’ recall bias. Only subscriber women and their husbands who made calls to the Aponjon consultation service in the past 3 months for maternal-, neonatal-, and infant health care-related queries were eligible for interviews. Exclusion criteria included callers who made calls for nonmedical purposes, who were younger than 18 years of age, and family members other than husbands. From the CRM database, we identified unique callers, prepared a sampling frame for two different types of callers (subscriber women or husbands), and systematically called every fifth caller. Each mobile phone was called a maximum of two times; we reached a caller almost 40% of the time (after making 81 call attempts to 45 phone numbers). Of the contacted callers, 18 agreed to participate in the study, and we arranged a convenient time for phone interviews. Two potential participants were excluded because they did not fit the entry criteria. Overall, 16 different families—eight women subscribers and eight husbands of subscribers—were recruited. Recruitment continued until data saturation was achieved, meaning that very little new relevant information was likely to be gained from further interviews with callers [44,45].

Recruitment of participant doctors was supported by Aponjon management. At the time of data collection, 16 medical doctors (12 females and 4 males) were registered for roster duties at the consultation service for morning, day, or night shifts. All doctors were contacted and informed about the research through Aponjon management. Among them, 11 doctors (9 females and 2 males) were available before or after their roster at the call center and were interviewed. The remaining doctors who could not be interviewed did not have a roster or were on leave.

We prepared interview guides for consumers (callers) and doctors which contained open-ended questions. Consumer participants were read the purpose of the research over the phone, and they gave verbal consent to participate and have their interviews recorded. Each phone interview lasted between 25 and 35 minutes. Participants were asked about their experience using the Aponjon hotline service—why they used it; the benefits, costs, and limitations of the service; and access to other health care providers. Participants’ socioeconomic information, such as age, education, monthly income, birth order, family size, occupation, and place of residence, was collected. Participants were also asked about how they individually accessed mobile phones, whether they had the capacity to receive or make calls, read, and send text messages without any help. Additionally, husbands (n=8) were asked how their wives accessed mobile phones.

All the doctors’ interviews were conducted face-to-face at the call center before or after their rosters. The doctors read the participation sheet before giving signed consent to participate and have their interviews recorded. Doctors were asked about their motivation to work in consultation service under mHealth initiatives, the type of advice they provided at Aponjon, callers’ expectations, and the benefits and barriers to a mobile-based consulting service. The interviews lasted between 35 and 50 minutes.

Author MA conducted all interviews in Bangla. All participants (callers and doctors) were given their rights to not answer any question or withdraw from the research at any point of time. Identities of all participants were masked by IDs.

**Figure 1:**
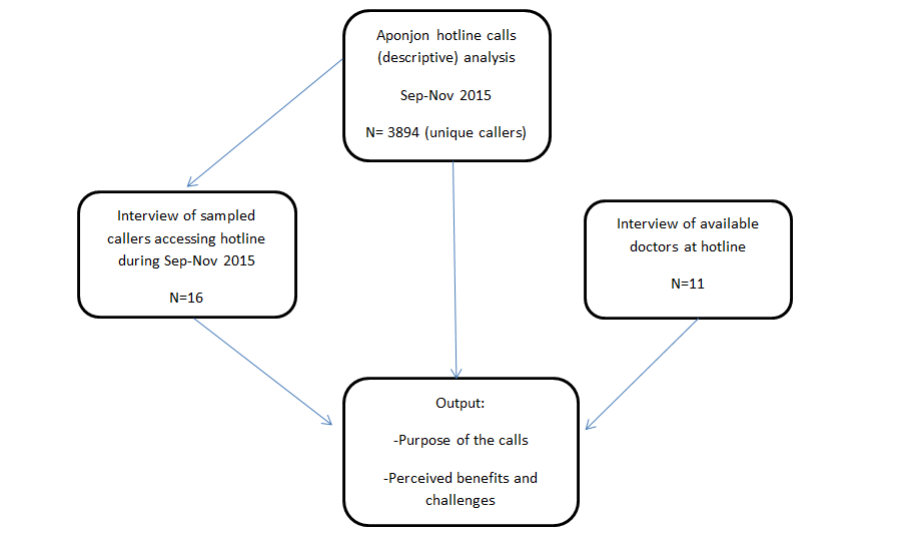
Mixed methods data triangulation plan.

### Data Analysis

The CRM database for the targeted period was analyzed with statistical software SPSS version 24.0 using a descriptive data analysis method. We ran frequency tests to describe patterns of call duration, type of callers, type of calls based on severity, and timing of calls; data were presented as frequencies, means, modes (where applicable), and percentages.

Professional transcribers and the author MA shared transcriptions of the interviews. All transcripts were converted to Bangla text. Author MA checked the recordings and transcripts. Transcripts were translated into English by author MA and then coded using Atlas Ti software for storage and to assist with analysis [35]. The transcripts were repeatedly read by authors to compare the doctors’ and clients’ experiences. A thematic analysis approach was used for identifying, analyzing, and reporting patterns (themes) within data [46]. Using this approach, a number of smaller subthemes [46] were identified inductively and then organized under two key metathemes identified by the authors: perceived benefits and perceived challenges. These two metathemes were developed after conducting a comparative analysis of concepts coded in different participant groups (participant callers and doctors). Codes and statements from participant groups were compared to assess positive and negative perceptions of the service [47], and were entered in a matrix. These similar perceptions from callers and doctors form the basis of the subthemes and are illustrated by quotes from both callers and doctors. A diagram (Figure 1) is provided on data triangulation for this study.

## Results

### Quantitative Findings

#### Who Were the Callers?

Approximately 3894 unique subscribers accessed the service during the 3 months for single or multiple consultations. There were three different caller groups based on who was making the calls from a single registered mobile number. There were 53.00% (2064/3894) who were subscriber women in the first group, who always made calls by themselves and consulted doctors directly. The second group contained callers other than subscriber women; 33.98% (1323/3894) of calls were made by husbands or family members who consulted doctors for subscriber women’s or babies’ symptoms. The third group, 13.02% (507/3894), were mixed callers (both subscriber women and husband or family members) from the same phone, inferring subscriber women shared mobile phones with family members.

The callers came from diverse areas across the country; call logs suggest that the service was accessed by callers from eight (8/13) districts in Dhaka, seven (7/11) districts in Chattogram, four (4/6) districts in Barisal, six (6/10) districts in Khulna, four (4/8) districts in Rajshahi, four (4/8) districts in Rangpur, one (1/4) district in Mymensingh, and four (4/4) districts in Sylhet division. In all, 68.36% (2662/3894) of calls came from rural households (Table 1).

**Table 1:** Caller type and location (N=3894).

Criteria		n (%)
**Who called**		
	Only subscriber women (pregnant/new mother)	2064 (53.00)
	Subscriber women and husband/family member	507 (13.02)
	Husband/family member	1323 (33.98)
**Place of residence**		
	Rural	2662 (68.36)
	Urban	1232 (31.64)

**Table 2:** Category of calls (N=5288).

Category of calls	Subject of queries about neonates and infants	Subject of queries about pregnant women	Subject of queries about new mothers	n (%)
General	Queries on general cold, fever, nutrition, neonatal and infant health care, development milestones, growth, play, immunization, and medicine; primary diagnosis of neonatal and infant illnesses	Queries on pregnancy health care, nutrition, fetal development, delivery, general cold, lifestyle, and medication; primary symptoms of pregnancy discomfort and illnesses	Queries on family planning methods, maternal health, reproductive health, and breastfeeding; primary symptoms of reproductive health and postdelivery complications	5081 (96.08)
Semiurgent	Neonatal and infant diseases, infection, birth-related injury, and developmental delays	Pregnancy and delivery-related danger signs	Maternal health issues	142 (2.69)
Urgent	Neonatal and infant illnesses, developmental problems, and birth-related injury	Pregnancy and delivery-related danger signs	Maternal health issues	65 (1.23)

#### Type of Calls

A total of 13,101 incoming calls were handled during this period. The minimum call duration for a complete conversation was recorded at 1 minute 40 seconds and the maximum at approximately 16 minutes.

The problems stated by the patients were triaged into nonurgent, semiurgent, and urgent categories by the doctors depending on the severity of symptoms. Complete data on the problem statement, advice, and related notes by the doctor were available for 5288 calls. From the available data, most calls were nonurgent (5081/5288, 96.08%); callers were seeking general advice on nutrition, growth, development on pregnancy, delivery, neonatal and infant health, and postpartum maternal health. The rest of the calls were made for semiurgent (142/5288, 2.69%) and urgent conditions (65/5288, 1.23%). Nonurgent calls were provided with self-care advice, prescriptions, appointment advice with local physicians, or referral to local facilities. Semiurgent and urgent calls required an emergency visit to local health facilities for labor, delivery, and newborn illnesses. A schematic guideline of the type of calls is provided in Table 2.

The call center received most calls for urgent or semiurgent conditions at night. Out of 207 such calls, 64.7% (134/207) calls came between 8 pm and 8 am (Table 3). Almost 56.1% (116/207) of late night calls were made by husbands and 43.9% (91/207) by pregnant women or new mothers.

**Table 3:** Morphology of emergency (urgent and semiurgent) calls (N=207).

Criteria		Urgent (n=65), n	Semiurgent (n=142), n	Total (N=207), n (%)
**Call time**				
	8 am-2 pm	18	15	33 (16.0)
	2 pm-8 pm	17	23	40 (19.3)
	8 pm-8 am	30	104	134 (64.7)
**Who called**				
	Pregnant woman	19	21	40 (19.3)
	New mother	5	46	51 (24.6)
	Husband	41	75	116 (56.1)
**Purpose of the calls**				
	Pregnancy or delivery related	38	63	101 (48.8)
	New born health	24	75	99 (47.8)
	Maternal health (postdelivery)	3	4	7 (3.4)

#### Call Interruption, Missed Calls, and Preferences

The CRM data for the study period indicated that 152 calls were ended by callers before the doctors could finish providing their advice, and 64 calls were disrupted due to a poor network. There were 342 missed calls reported in the call notes in which callers cut the call after one ring.

Special notes suggest that there were three incidents in which the subscriber called back to update on prognosis, one incident in which the female subscriber asked to talk to a female doctor, and one incident in which the female subscriber wanted to talk to the doctor she previously consulted.

### Qualitative Findings

The callers, eight women and eight husbands representing 16 families (Table 4), came from diverse geographic locations in Bangladesh; they were a mix of rural (12/16) and urban residents (4/16). Most of the participating families were middle income. The women callers (Table 4) were mainly younger than 25 years (5/8); two were in their late teens (18 and 19 years). Educational levels differed between families of female callers and male callers. Female callers and their husbands had higher levels of education (most of them had completed higher secondary education) than male callers and their wives. Women callers reported more autonomy in accessing mobile phones (such as making and receiving calls, texting, and owning a personal phone) than women whose husbands called Aponjon.

**Table 4:** Background information of participants (Aponjon subscribers) (N=16)

Characteristics			Caller women n (n=8),	Caller husband (n=8), n
**Age of participants (years)**				
	<20		2	0
	20-24		5	1
	≥25		1	7
**Access to Aponjon messages**				
	Regular		7	5
	Sometimes		1	2
	Never		0	1
**Type of messages received**				
	Voice		8	5
	text		0	3
**Who received Aponjon messages**				
	Husband		0	3
	Wife		8	0
	Both		0	4
	Never received messages		0	1
**Aponjon subscriber woman possesses a functional personal mobile phone**				
	Yes		8	2
	No		0	6
**Mobile phone literacy of Aponjon subscriber women**				
	**Can receive and make calls**			
		Yes	8	5
		No	0	3
	**Can send/read text messages**			
		Yes	8	3
		No	0	5
**Educational qualification of subscriber women/wives**				
	None or primary education		0	3
	Junior secondary education		2	0
	Secondary school or higher		6	4
	Don’t know		0	1
**Educational qualification (husbands)**				
	None or primary education		0	3
	Junior secondary education		0	0
	Secondary school or higher		6	5
	Don’t know		2	0
**Family monthly income (BDT)**				
	<10,000		0	1
	10,000-50,000		8	6
	>50,000		0	1
**Occupation of woman**				
	Homemaker		6	8
	Teacher		1	0
	Student		1	0
**Occupation of husband**				
	Overseas work		4	0
	Unemployed		0	1
	Small or large business		3	5
	Other paid jobs		1	2
**Family type**				
	Nuclear		1	1
	Extended		6	7
	Stays with parents (husband overseas)		1	0
**Number of children**				
	Expecting first child		2	0
	1		3	5
	2		3	1
	3 or more		0	2
**History of miscarriage/stillbirth/abortion**				
	Miscarriage/abortion		1	1
	Child death (obstructed delivery)		1	0
**Division**				
	Dhaka		5	3
	Rangpur		1	0
	Rajshahi		0	3
	Chittagong		2	0
	Khulna		0	2

**Table 5:** Demographics of participant physicians (N=11).

Characteristics		Physicians
**Gender, n**		
	Female	9
	Male	2
**Age (years)**		
	Median (IQR)	28 (3)
	Mean (SD)	28 (2)
	Range	25-32
**Duty hours per week at call center**		
	Median (IQR)	21 (7)
	Mean (SD)	18.9 (4.7)
	Range	11-28
**Experience with Aponjon service, n**		
	<3 months	2
	>6 months	1
	>1 year	8
**Experience after graduation (years), n**		
	1-3	2
	3-5	5
	>5	4
**Special training, n**		
	Gynecology	4
	Pediatrics	5
	Medicine	2

Unlike a previous study [32], in which all Aponjon subscribers were enrolled in the service through health workers, only one-third (5/16) of participants were recruited to the service via this method. The remaining participants enrolled via a nurse or paramedics (6/16) during antenatal visits at hospitals and by self-enrollment (5/16) after seeing advertisements on television or in the newspaper.

All participating doctors (n=11) were medically trained and had received training in gynecology and pediatrics. The doctors were young with few years of experience after graduating from medical school (Table 5). In addition to working at the call center, they also worked at private and public health facilities.

### Theme 1: Perceived Benefits

#### Access

All callers were satisfied with the consultation service because they could access a trained doctor any time they wanted. Women who were able to make calls on their own found the service convenient to access medical advice without relying on male members to organize a visit to a general practitioner (GP). Women had a sense of relief because they could discuss their pregnancy stages, newborn care practices, and symptoms of illnesses with Aponjon doctors. One teenaged new mother accessed the service frequently to discuss newborn care and disease symptoms. The doctors also reported receiving calls from young women and their husbands. The customer-centric approach of the call center encouraged doctors to use friendly greetings, to listen to queries with patience, and to provide practical advice. There was a policy that the “customers hang up calls, not the doctors,” which encouraged callers to contact the service without any reservation. The doctors reported that they could allocate more time to patients’ problems at call centers, which was impossible at crowded outdoor services at health facilities. Some doctors thought mobile consultation encouraged shy women to discuss their reproductive health. One doctor reported:

There are women who do not want to visit a doctor; they feel shy. But since we don’t see the patients face-to-face some women think their privacy is protected and they share their private problems with us. I have received many calls like that. When we receive the calls, we tell our name like “I am Doctor X.” Women who use the service regularly remember our names and sometimes would ask for the same doctor

Doctor 6

One anxious pregnant woman illustrated how the service gave her ready access to reassurance and information:

I had a miscarriage before this one, so naturally I am a bit tensed all the time and I want to clarify the symptoms with a doctor every now and then. I feel very low when my Gyne doesn’t pick up the call or if she hurries through my calls. She won’t suggest anything on phone, and asks me to visit her chamber right at that moment, which is not always possible especially if it is late at night. So, I call Aponjon doctors whenever I am feeling unwell. They listen to me patiently, and they advise me whether my conditions are severe. If they ask me to see my Gyne or visit a hospital for a check-up, I do that without any delay.

Caller 8, rural pregnant woman

The service was convenient for rural people living in remote areas who otherwise had two options: travel for several hours to discuss a health-related concern with a qualified doctor at a facility or consult the local drugstore salesman who has no professional qualifications. Husbands who were unable to allocate time, money, or leave from work to accompany their wives to health facilities thought a mobile phone-based consultation service was a convenient option for initial diagnosis. Sometimes husbands would organize phone calls so that their wives could talk to the doctors. One husband stated:

I have been accessing this service for almost a year now since the beginning of my wife’s pregnancy. I often make a call to the service, I even called yesterday. It is difficult to find doctors (for a visit) all the time, and I work in the [...] department. So, I cannot always make time to take my wife to see a doctor. It takes a lot of time-waiting in the queue, traveling, and the fee [private doctor’s] is between 300 and 400 taka [US$ 3.56 to 4.74] which is costly for us. So, I like this service for primary consultation. It’s very useful as we can call anytime and get good advice.

Caller 3, rural father

The doctors reported receiving calls from both disadvantaged rural community and urban households, although more came from rural Bangladesh. This is not surprising as Aponjon recruited a large proportion of its client base through rural community health workers. The doctors considered their job rewarding and felt that they were useful in availing themselves to rural patients.

I have received calls from poorest of the poor (rural) to bankers (urban). But most of the time the callers are low-income people who are located in very remote places and live quite far away from health facilities or upazilla [subdistrict] health complexes. I think this [call from low-income households in rural areas] is because most of the recruitments to the service were done by NGO health workers. They [patients] are able to know where they have to go or what to do by making a call to us. Most of these calls are nonurgent, but we get urgent calls too mostly at night. I feel very happy at the end of the day that I can be of some help to rural people who need it most.

Doctor 11

The option to talk to a doctor at the call center regarding maternal and newborn health was advertised on TV channels and newspapers, which interested some participants to enroll in the service. Availability of female doctors at the call center encouraged women callers to access the consultation service; some participant women would rather wait for female doctors to be rostered on if the problem was not acute. The service was useful to clients at night time when visiting a doctor was not possible. The doctors reported that they were getting increased calls regarding problems that required urgent attention mostly late at night. One such incident noted by a participant woman:

We were very worried one night when my son [infant] became ill. My husband contacted Aponjon. At that time the doctor gave us a good solution which improved his condition. In the morning we took him to a hospital.

Caller 6, rural mother

#### Trust

Participating callers explained that they considered Aponjon’s consultation service to be a trusted source of medical information and clinical advice. Aponjon doctors confirmed that they graded the severity of the illnesses after careful consideration of the symptoms. Often, on request of the women who made the call, they motivated reluctant family members to seek treatment from health facilities. The doctors reported that they received many calls in which callers sought a “second opinion” from them regarding cesarean delivery or other treatment at hospitals.

One doctor stated:

I once referred a mother for further investigation to nearby medical hospital; her baby suffered from brain injury during delivery which required proper treatment to avoid impairment. Doctors at Mymensingh Medical College Hospital referred her to Centre for Rehabilitation of Paralyzed in Savar, specialized for this treatment. But the mother was in a dilemma and called me from the hospital for assurance.

Doctor 9

Aponjon’s mobile phone-based consultation service offered a convenient medium to families through which they could demystify cultural myths and practices with someone who they believed to have superior knowledge and medical training— *pash kora daktar* with a Bachelor of Medicine, Bachelor of Surgery (MBBS) degree. Doctors reported receiving calls from women, their husbands, and even mothers-in-law who sought advice on the care of pregnant women, lactating mothers, and newborn babies. Mothers-in-law are powerful household members who have considerable influence on the birthing and health care practices of their daughters-in-law. For example, some female callers (Aponjon subscribers) rang on behalf of their mothers-in-law to inquire about commonly practiced rituals, such as rubbing mustard oil on the newborn baby’s umbilical stump. “I could overhear the mother-in-law asking her [daughter-in-law] to ask me these questions,” said a doctor. Some anxious mothers-in-law called to inquire about nutrition for pregnant women, how to increase the production of breast milk, or the illness symptoms of the mother and infant. The doctors supported new fathers, who then felt empowered to resist the traditional practices of elderly family members. One new father reported:

The doctor told me not to feed newborn babies mustard oil. He also told me that we should not perform shak dewa [vapor treatment] around umbilical stump of newborn baby. That was so helpful! You know how murubbi [elderly family members] ask us to do that. I prevented them [from performing on my newborn]. Instead, we applied a powder [chlorhexidine] which the doctor told me over phone.

Caller 2, rural father

Satisfied husbands wanted to expand the service beyond the current scope (pregnancy, postpartum maternal health, newborn and infant health) and expressed an intention to use the consultation service for both men’s and women’s health in all age groups.

#### Cost

The service price (2.3 taka per minute or US $0.03) did not seem to be a barrier to access direct consultation service for the participant callers; they were willing to pay for the consultation service. As this participant suggests, the cost of the service is lower than the cost of attending a face-to-face medical consultation:

We [he and his wife] practically are bokolom [illiterate]. They send us recorded messages, we both listen.The consultation service with doctors through mobile phone is very helpful for us. They give us direct advice. I cannot close my shop and spend time traveling and waiting to see a doctor all the time. Every day I sell products worth 1000 to 1200 taka [US $11.85 to $14.22], my profit remains 100 to 150 taka [US $1.19 to $1.78] and with that money the four of us have to survive. How can I close my shop for a moment, tell me?...I don’t find the service expensive. If you [Aponjon] need money, tell me, I will flexi [mobile cash] some money [to Aponjon], no problem.

Caller 1, rural father

Another participant weighed the cost against the value of a child’s health:

I don’t think the charge [for consultation service] is too much. You can’t ignore your child’s health, right? I visit my baby’s pediatrician quite often who is available 2 days a week and located only 10 to 15 minutes away from my home. But still I think there are so many queries I should ask, and I prefer calling Aponjon. I don’t have to book tickets and go through all the hassles [at hospitals] for phone consultations.

Caller 16, urban mother

These quotations suggest that participants made thoughtful decisions about cost by considering a number of elements.

#### Raising Awareness and Assisting with Decision Making

Some participants found the consultation service to be very useful besides the weekly messages they received; they had the scope to discuss their confusion regarding any messages with the doctors. Doctors reported receiving calls from patients requesting clarification on weekly messages. Some participants reported problems with receiving messages due to a network problem or inadequate balance issues in their mobile phones; they thought calling the doctors was always a good option to catch up with what they had missed. One participant husband stated:

I tell my friends about the service. We get shundor shundor [nice] messages every week from this service. My wife and I both receive the messages and have learnt new information. The messages have made us conscious about a lot of things. And then whenever we have a query regarding our baby I straightaway call and talk to the [Aponjon] doctor who is again helping us with proper advice and (sometimes) medication. I can share everything [with the doctor] which is a great thing.

Caller 9, urban father

Interviews with both the callers and doctors suggest that doctors were able to raise awareness about the dangerous signs of pregnancy, delivery, and neonatal illnesses. Doctors reported a common “inertia of families toward seeking help from outside” and in response worked to educate callers on a range of issues, such as routine antenatal checks, counting of fetal movements, and symptoms of eclampsia (one of the most prominent causes of maternal mortality in Bangladesh) [4].

My wife is expecting our fourth child...it was not a planned pregnancy...our first child is an adult. Never before [for other pregnancies], my wife had an ultrasound, but this time when Aponjon doctor told us to do that, we performed the test. [Now] we know the baby is okay [inside the womb] and we also know the expected date of delivery.

Caller 17, rural father

The doctors reportedly interfered with callers’ decisions to seek care from untrained providers, such as the local drugstore salesman or traditional healers. One incident reported by a doctor:

Once a newborn baby was having convulsions, and the family decided to take the baby to a Kabiraj [nonformal provider] to perform jhar-phoonk [rituals believed to have magical effects] which is harmful [to the baby]. The father of the child called Aponjon...I asked him to remove the patient [child] to a hospital immediately. I explained the illness of the child and he understood it.

Doctor 8

A telephone consultation with doctors during an emergency such as a prelabor rupture of a membrane (water break) or prolonged labor can help family members make a safer decision and ultimately save the life of the mother and the unborn child. One such incident reported by a doctor magnifies the need for such guidance during emergencies:

My shift was almost over that night when the husband called. He was confused as the village doctor gave saline [at home] and left saying that in the morning everything will be fine [a vaginal delivery]...I suspected it could be a case of an obstructed labor and instructed him to remove his wife to a hospital that night...We later received a call from the husband that an emergency cesarean procedure had saved his wife and baby. The doctors at the hospital said he was just in time.

Doctor 4

#### Authentic Prescribing

Callers from both urban and rural households reported purchasing medicine for simple or severe complications from local drugstores. Drugstore salespeople are typically unqualified allopaths with no formal degree, having learned their trade via apprenticeship with a qualified or unqualified practitioner [48]. Medication dispensing is often negotiated depending on the severity of the complaint, the economic situation of the patient, and the range of available medicines [48]. Aponjon doctors reported alerting callers to the inappropriate use of medications. Additionally, the service has the capacity to provide callers with prescriptions for over-the-counter drugs if required. The doctors took great care to spell the names of prescribed medicines correctly. Usually, if the patients were unable to follow the doctor’s instruction, they sought help from a literate person in the family to write down the names of medicines. Sometimes patients would call the doctors from the pharmacy so that the doctors could talk directly to the salesperson about the particular medicine. One doctor reported:

Patients expect that we will give them solutions for everything through medicines. They want names of medicines which they can purchase from pharmacies and get cured without visiting health facilities. We do not support that. As we cannot see the patients, we provide advice and prescribe over-the-counter drugs if necessary. We ask them to notify us about their condition within a few days. If the condition persists, we ask them to visit local health facilities.

Doctor 5

One participating mother explained the importance of receiving trained medical advice:

My son is only 4 months. There is no pediatrician in my area. So, I saw a provider who gave three medicines. Then I called to Aponjon and told the doctor the name of the medicines. The doctor asked me not to follow those medicines as my son is too young and insisted to see a pediatrician. I did not give the medicine [and] saw a pediatrician from the district hospital.

Caller 4, teenaged rural mother

### Theme 2: Perceived Challenges

#### Logistical Issues

The doctors and callers both mentioned problems with consultations when the network dropped out. For example, an anxious husband reportedly was desperately trying to contact a doctor when his wife’s labor progressed, but “the calls failed again and again.” Apart from network issues, the call center line can be busy forcing callers to try several times:

Overall, I like the service. For consultations, I spend approximately 20 taka [US $0.24]. Sometimes getting the line through to the doctors takes time. There is network problem, and then often the lines are busy. But still, I make the calls.

Caller 12, rural mother

#### Equal Access

As noted previously, most participants considered the service call rate (2.3 taka or US $0.03 per minute) “cheap.” However, we do not know if the service was inaccessible to low-income households. Even among households who could afford the service, doctors reported that the quality of consultation was compromised when clients kept the consultation time short to manage the expense. One doctor reported:

Usually, most patients want us to explain the cause of their symptoms and often the discussion can lead up to 15 to 20 minutes depending on the patient’s query. Sometimes the network can be problematic; the phone line can get disrupted. Clients call back and finish the discussion. But some clients cannot afford long conversations; they are in a rush. They don’t even want us to greet them, they straight jump to their problems and want us to give them a solution in the quickest possible time. They tell us “hurry up” [taratari bolen]. Sometimes before we have finished, they just hang up. We cannot even record their case history properly.

Doctor 1

#### Visual Examination

The doctors reported that their foremost challenge in virtual consultation was the difficulty diagnosing symptoms without the ability to conduct a visual examination. For example, the doctors could not verify whether a person actually had “jaundice.” They often had to innovate ways to understand the temperature of a “feverish” child over the phone if the parent did not have a thermometer or was not confident in using one. Doctors sometimes faced challenges understanding patients’ colloquial language and had to spend time seeking clearer descriptions of symptoms. Similarly, prescription of over-the-counter drugs was difficult without knowing the baby’s weight and checking for other fatal illnesses such as pneumonia. As one doctor said, “We cannot always rely on how they explain the symptoms. What is a ‘mild’ condition according to them can be ‘severe’ in our diagnosis.” Consequently, referral to a health facility for diagnosis was a common outcome of calls.

#### Referral and Follow Ups

Aponjon doctors identified the lack of connection between the consultation service and available care and emergency services as a key weakness in the service. The doctors were unable to follow up with patients about referrals and expressed concerns about whether patients could navigate the right care at the right facilities. Indeed, according to patient callers, delay in finding the right care often varied depending on the distance between their residence and a quality health facility, their financial situation, and social connections. For example, a female rural participant had to organize overnight stays at her parents’ house in the city when she needed to visit a pediatrician at a public hospital. Similarly, another participant, a first-time father, had relocated his pregnant wife from his ancestral village home to his residence near his workplace because his work colleagues had alerted him to the availability of quality emergency obstetric services, which were hard to find in rural areas. A doctor also reported his experience of receiving patients at urban facilities with much worse symptoms due to a significant delay in finding the right care:

I have seen parents from remote areas seeking treatment at private hospitals for neonates with severe illnesses. They travel from very remote areas to the city. The neonates get admitted at neonatal intensive care [at the private hospital]. What can those [rural] parents do? The local [public] facilities probably don’t have the support.

Doctor 2

The service also lacked a policy to follow up on how patients were complying with medical advice at home regarding issues such as prescriptions. Doctors stressed the need for a database to track patients’ case histories, prognoses, and referral outcomes.

## Discussion

We investigated the access, acceptability, usability, benefits, and challenges of a mHealth consultation service in Bangladesh. Our research is novel because we have combined analyses from both call log data and perceptions of patients and providers. We analyzed call center data on frequency, duration, urgency level, and advice sought by subscriber callers of a mHealth consultation service who also accessed the regular messaging service. We also conducted in-depth interviews with subscriber callers and doctors of the mHealth consultation service regarding their perceptions, and the perceived benefits and barriers in using such a service for maternal, neonatal, and infant health. Our analyses of call log data and qualitative interviews are complementary. Overall, our findings suggest that the consultation service was trusted among callers and contributed to significant changes in health care-seeking behavior (Table 6). The call log data suggest that more than half the calls were from subscriber women (pregnant women or new mothers) and that majority of all callers were rural residents. This suggests that mobile phones may help address culturally determined gender norms in Bangladesh by enabling “shy” women to discuss their personal health issues with female doctors who are hard to find in rural communities [2,49]. The service may lead to a paradigm shift in how women seek health care. Young married women in low-income households are generally guided by their husbands and mothers-in-law, who may deter them from accessing skilled maternal health care services [49,50]. This research shows that some women were reluctant to visit maternal health care services due to distance, cost, fear of hospitals, privacy concerns, unavailability of female physicians in local facilities, need for a male chaperone, and being refused approval of husbands or mothers-in-law who thought the symptoms were not so severe or could be cured at home [9,49]. We find that Aponjon’s remote consultation service has the potential to empower women in rural communities to discuss their queries regarding maternal, neonatal, and infant health. Findings from qualitative interviews suggest almost all participant caller women and their husbands found the consultation service convenient, accessible, and cost-effective for any medical advice and primary diagnosis. Trust in the service encouraged husbands to make phones available to their wives to consult Aponjon physicians; a step toward reducing the gender gap in women’s ownership and access to phones in Bangladesh [51]. Our findings provide useful evidence for mHealth implementers in similar socioeconomic and cultural settings of other South Asian countries, such as Nepal, India, and Pakistan [52,53].

This study suggests that the mHealth comprehensive system of messages and the consultation service is moving in the right direction to respond to the growing consciousness of maternal and newborn’s health and aligns well with other national interventions to improve infant health and well-being [3,6]. Talking to a doctor by phone is a plausible solution for subscribers with limited mobile phone literacy who may find it difficult to retrieve missed IVR messages [20,58].

Remote mobile consultation may improve people’s decision making during emergencies and reduce delay in reaching health facilities [10,59]. Call center data and qualitative interviews suggest that the real-time advice was instrumental in helping families understand the graveness of symptoms and encouraging them to seek immediate help, especially at night when visiting a doctor is technically impossible. The Aponjon consultation service was able to influence mothers-in-law and husbands, particularly, in advising the family to attend a medical service rather than a traditional healer.

**Table 6:** Identified benefits and ways forward.

Benefits	Way forward
The service was available 24*7, especially at night	Since there is a need for consultation late at night when physical visits to doctor is not possible, Aponjon service could open the consultation service to non-Aponjon subscribers (pregnant women and new mothers)
Women could talk with female doctors	During enrollment, community health workers or call center agents should inform women about the option to talk to a female doctor regarding symptoms and illnesses
Consultation service was cost-effective because it saved time, money, and absence from work	Each consultation should be documented in electronic medical records to improve consultation time and quality in following sessions; the service may include electronic prescriptions which patients can access through Aponjon apps [54]
Advice of doctors relieved pregnant women and new mothers of anxiety; dispelled myths and wrong practices	The service may extend counseling services to women who experienced miscarriage, stillbirth, and postpartum depression with accredited psychologists [55]
The service helped families to understand graveness of symptoms and making decisions during emergencies	The consultation service needs to improve referral system linked with local facilities so that the families may seek treatment at local facilities without delay [56]
Doctors were instrumental in raising awareness about unnecessary medication from unauthorized personnel	Drug Procurement Act [57] should be strengthened so that purchase of antibiotics and other medicines without a prescription is prohibited

**Table 7:** Identified challenges and recommendations.

Challenges	Recommendations
Consultation gets disrupted due to network problem	There is no immediate solution for such disruption, while continued investment in infrastructure of telecommunication network will improve the quality of calls in future
Talk time is expensive for the poor	Aponjon may create a toll-free option for selected communities where subscribers have low wealth index [14]; Aponjon may also explore other ideas such as mobile cash transfer to marginalized customers for talk times [36]
Doctors struggle to diagnose certain symptoms without visual examination	In this context, a training program of local providers, such as village doctors, community health workers, and *Dai* s, should be revived. Since the local providers are the foremost people to be contacted during delivery or any other illnesses, their explanation of symptoms could provide a realistic picture of the condition of the patients to the doctors over phone [14,58]
Referrals of the doctors to seek treatment at health facilities or a specialist service is not specific	A proper integrated system plan is required; the consultation service requires to be linked with existing local facilities; a smooth connection for emergency transport needs to be organized between consultation service and existing facilities; data interoperability between service centers and a national patient database is warranted [61-66]
The consultation service did not follow up patients on medication and referrals	Toll-free follow-up calls and reminder text messages to patients regarding appointments and medication need to be integrated in the service [67]

Despite several benefits recounted by callers and doctors regarding the consultation service, we find that the service has potential to improve and thus have included recommendations (Table 7). In terms of equity, the consultation service may still be too costly for low-income households who reportedly kept conversations short to minimize talk times. Under such circumstances, the current pro-poor policy of the service [31] is not effective in securing access for low-income households free of charge, pushing them to rely on unqualified health professionals for low-cost treatment [2,3]. Customized approaches of previous interventions, such as a toll-free communication line [14] or mobile cash transfer [36], for “talk times” remain options to keep the service affordable to low-income households. The service may build on the successes of the national maternity voucher system in selected districts by increasing utilization of delivery facilities in marginalized communities and providing similar support to subscriber women from low-income households to be able to receive discounted MNCH services at local facilities [60].

Our study findings suggest that the doctors provided triage based on the severity of symptoms, but lacked an operational referral service to support their decisions, leaving patients to their own resources to find a physician or health facility. A study in rural India suggested there was an increase in utilization of MNCH services and growing demand for facility-based services by pregnant women after sessions of mHealth-supported counseling service by community health workers [68]. To respond to similar circumstances, we propose that an m-referral linked with the consultation service is essential to increase the efficacy of Aponjon service. In fact, a well-functioning mHealth service is incomplete without a proper referral service and is undeniably reliant on the capacity and readiness of the existing health system to provide quality care to citizens [69]. The government is progressing major reforms in the health system to ensure access to universal health coverage by all citizens [3], and m-referral can play a pivotal role in these reforms by offering patients real-time information on existing health care and referrals to quality medical care. An integrated referral system could provide routine toll-free follow-up calls to referred patients to ensure patient well-being and adherence to treatments [67]. In particular, the service could assist users from rural low-income households navigate the health system and potentially avoid the ingrained practice of bribery by poorly trained staff and *dalals* (middlemen) in public health facilities [2,70]. Referrals from Aponjon to specific destinations would require thorough information on the existing capacity of local facilities. For example, adherence with a referral advice was high in a recent pilot study [56] in Bangladesh in which the efficacy of an emergency transport system for referring sepsis patients to nearby local facilities was tested, after assessment by a call center with the aid of a geographic information system. Although this study was conducted among a small population, lessons from this study may guide the Aponjon consultation service in establishing linkages with local health facilities for referrals. The referral system also needs to build a framework for electronic medical health records and a robust platform for data interoperability between consultation services and health facilities, which will allow transfer of patient data such as symptoms, primary assessment, past medical history, findings, treatment, medication, care plan, special notes, and immunization [61,62]. The need for a national database for patient electronic health records is indispensable to accommodate heterogeneous patient data from different sources. This will improve the referral system, treatment, and allocation of medical professionals accordingly, and enhance the prospects of longitudinal studies evaluating disease causation and prevention [63-65]. Due to growing concerns about security breaches of electronic records worldwide, the system will need to ensure the protection of patient identity, maintenance and monitoring of firewalls, training of medical staff on confidentiality and ethics, and access of data to appropriate personnel only [66].

The doctors expressed difficulties in understanding the symptoms of patients who could not express themselves well over the phone and felt the necessity for a visual examination. The need for visual examination may be mitigated by images sent from smartphone apps by patients [71]; however, this may not be a practical solution in Bangladesh where smartphones are still not a cheap option for rural disadvantaged households [72], and maneuvering a smartphone requires soft skills beyond the limited ability to receive or make calls. We suggest that local resources, such as the village doctors, *Dai* s (traditional birth attendants), or community health workers, the first people to be contacted during emergencies, may help explain the symptoms to remote doctors and arrange emergency referrals in rural areas [14]. Capacity building of local resources is a prerequisite for this model to work and requires a well-structured and coordinated national capacity building program. mHealth-linked call centers established between village doctors and professional medical doctors were suggested as a model to build the capacity of semiqualified professionals in Chakaria, Bangladesh [58]. Semiqualified or unqualified village doctors account for approximately 80% of local providers in Bangladesh and are known for inappropriate medication, polypharmacy (prescribing five or more medicines in one prescription), and inappropriate injections at the “patient’s demand” [73]. mHealth-linked professional training of village doctors could improve remote diagnosis and adherence to an appropriate prescription policy in Bangladesh [57]. We could benefit from mHealth doctors’ advocacy in raising awareness among callers about appropriate pharmaceutical prescriptions and use, thus contributing toward a change of behavior in consuming self-medicated antibiotics purchased without prescription at local drugstores [74]. Additionally, a national strategy and initiatives to train traditional *Dai* s and community health workers should also be revived [3]. Evidence from another study that showed improved performance of community health workers in screening high-risk patients with the aid of mHealth algorithms and training modules may be adopted in programs targeted to build the capacity of community health workers [68,75].

Our study is limited by a number of factors. First, the sample size for interviews was small, which may impact the generalizability of our findings; however, data triangulation (analysis of call log data) was adopted to minimize the limitation [45]. Second, due to the systematic sampling of interviewees from the call log list, low-income households are underrepresented in the qualitative study [76]. Third, we had access to limited data in the call list; a dataset containing clients’ age, education, occupation, economic status, and follow-up results could improve analysis on adoption of mobile phone consultation service among women. Fourth, our research does not address sustainability issues such as operational costs, technological challenges, and revenue generation by the call center. This is vital information for understanding the possibilities of service expansion and escalation. A future quantitative study including a cost-effectiveness analysis, willingness to pay among callers, and the effects of doctors’ remote consultations on health outcomes and referrals is warranted.

Despite the limitations, our study is significant for a number of reasons. We presented experiences of two groups of families—one where women could access the service themselves and the other where women had to rely on their husbands—to understand how gender, autonomy, and mobile phone literacy play a role in women’s utilization of telehealth care. These are important considerations when designing future mHealth interventions. We advocate that the inclusion of husbands in health interventions would accelerate women’s access to health care for positive outcomes. Our study reports on the experiences of doctors who recounted the benefits and challenges of remote diagnosis in comparison to health facilities. The call log data inform us about the call time, frequencies, level of urgency, and access of subscribers. These insights offer the opportunity to improve our understanding of the benefits and challenges of mHealth as services develop and expand across Bangladesh and other low-income countries.

Mobile phone consultations or telehealth for maternal, neonatal, and infant health care are a feasible primary health care activity to increase access to professional medical advice, especially in remote areas of Bangladesh. A remote consultation service may influence some of the cultural and economic factors that delay maternal and child health care-seeking practices. A collaborative well-designed referral system connected with specialists and emergency MNCH services may improve health outcomes for urgent conditions. A central government-run database to collect heterogeneous patient data from public-private initiatives will ensure prompt management of emergency referrals generated by mHealth services. We recommend the systematic use of quantitative surveys to understand health care-seeking behaviors and health outcomes of users, as well as trials on an integrated referral system between call centers and health facilities.

## Abbreviations

BDT: Bangladeshi taka (currency)
CRM: customer relationship management
GP: general physician
IVR: interactive voice responses
MNCH: maternal, neonatal, child health care
